# Genetics of narcolepsy

**DOI:** 10.1038/s41439-018-0033-7

**Published:** 2019-01-08

**Authors:** Taku Miyagawa, Katsushi Tokunaga

**Affiliations:** 1 Sleep Disorders Project, Department of Psychiatry and Behavioral Sciences, Tokyo Metropolitan Institute of Medical Science, Tokyo, Japan; 2 Department of Human Genetics, Graduate School of Medicine, The University of Tokyo, Tokyo, Japan

**Keywords:** Risk factors, Genetic markers

## Abstract

Narcolepsy is a term that was initially coined by Gélineáu in 1880 and is a chronic neurological sleep disorder that manifests as a difficulty in maintaining wakefulness and sleep for long periods. Currently, narcolepsy is subdivided into two types according to the International Classification of Sleep Disorders, 3rd edition: narcolepsy type 1 (NT1) and narcolepsy type 2 (NT2). NT1 is characterized by excessive daytime sleepiness, cataplexy, hypnagogic hallucinations, and sleep paralysis and is caused by a marked reduction in neurons in the hypothalamus that produce orexin (hypocretin), which is a wakefulness-associated neuropeptide. Except for cataplexy, NT2 exhibits most of the same symptoms as NT1. NT1 is a multifactorial disease, and genetic variations at multiple loci are associated with NT1. Almost all patients with NT1 carry the specific human leukocyte antigen (HLA) allele HLA-DQB1^*^06:02. Genome-wide association studies have uncovered >10 genomic variations associated with NT1. Rare variants associated with NT1 have also been identified by DNA genome sequencing. NT2 is also a complex disorder, but its underlying genetic architecture is poorly understood. However, several studies have revealed loci that increase susceptibility to NT2. The currently identified loci cannot explain the heritability of narcolepsy (NT1 and NT2). We expect that future genomic research will provide important contributions to our understanding of the genetic basis and pathogenesis of narcolepsy.

## Introduction

Narcolepsy was first described by Westphal in 1877 and named by Gélineáu in 1880. After rapid eye movement (REM) sleep was discovered in 1953^1^, several investigators studied sleep onset in patients with narcolepsy. Although healthy individuals typically enter their first REM sleep approximately 90 min after falling asleep, patients with narcolepsy frequently go directly into REM sleep at sleep onset^2^. A malfunction of the mechanisms that regulate REM sleep can explain some of the symptoms of narcolepsy. Narcolepsy has no known cure at present. Although its symptoms can be managed with appropriate treatment, lifelong treatment is required for most patients. Currently, two forms of narcolepsy are recognized: narcolepsy type 1 (NT1) (previously termed narcolepsy with cataplexy) and narcolepsy type 2 (NT2) (previously termed narcolepsy without cataplexy)^3^. NT1 is a typical type of hypersomnia that is characterized by excessive daytime sleepiness (intolerable sleepiness in the daytime), cataplexy (sudden loss of muscle tone in response to strong emotion), and pathological manifestation of REM sleep, which includes hypnagogic hallucinations (dream-like experiences occurring at sleep onset), sleep paralysis (feeling of the inability to move while falling asleep), or sleep onset REM sleep (appearance of REM sleep within approximately 5–10 min after sleep onset). The onset of NT1 usually begins between the ages of 10 and 20 years and affects 0.16–0.18% of the general population in Japan^4,5^ and 0.02–0.06% of the populations in the United States and Europe^6^. The lowest prevalence rate appears to be in Israel (0.0002%)^7^. The concordance rate in monozygotic twins for NT1 is approximately 20–30%^6,8^. Approximately 1–2% of first-degree relatives of a person with NT1 are affected by the disease. The relative risk for first-degree family members of patients with NT1 is approximately 10- to 40-fold higher than that in the general population^6,9^. Therefore, NT1 is a complex disease with both genetic and environmental risk factors. NT2 has the same symptoms as NT1 except for cataplexy. An epidemiological study found that the prevalence of NT2 was 36% of that of NT1, corresponding to a point prevalence of 0.02%^10^. Fewer studies have been conducted on the epidemiology of NT2 than on NT1. Recently, high-throughput technologies have been developed to efficiently obtain genotype data in a genome-wide manner and have identified susceptibility loci for NT1. In addition, genetic analyses of NT2 have progressed gradually. In this review, we focus on genetic research that has elucidated the pathogenesis of narcolepsy (NT1 and NT2).

## Orexin (hypocretin) deficiency in patients with NT1

In 1999, two independent groups revealed the pathogenesis of narcolepsy in animals^11,12^. Orexin A and orexin B (also known as hypocretin 1 and hypocretin 2, respectively) are neuropeptides that regulate arousal, wakefulness, and appetite and are produced exclusively by neurons in the lateral hypothalamic area^13,14^. A disruption in the canine orexin receptor-2 gene causes autosomal recessive canine narcolepsy^11^. In addition, prepro-orexin knockout mice exhibit narcolepsy-like behaviors and electroencephalographic activity^12^. In humans, the orexin A level is severely reduced or undetectable in the cerebrospinal fluid (CSF) of approximately 90% of patients with NT1^15^. NT1 is characterized by a low orexin A level (<110 pg/ml) and cataplexy.

In contrast, patients with NT2 have normal orexin A levels and do not exhibit cataplexy^16^. Thus, measurement of orexin A in the CSF is an important diagnostic tool. Variant screening for prepro-orexin and orexin receptor genes was performed in patients with NT1. However, unlike canine narcolepsy, the patients did not have disease causative variants in the prepro-orexin and orexin receptor genes except for one severe early-onset case. In contrast to the monogenic canine narcolepsy model, narcolepsy in humans is sporadic in most cases and is caused by multiple genetic and environmental factors. Although the link between orexin dysfunction and narcolepsy is consistent in both humans and canines, the mechanisms underlying the dysfunction are different.

## Strong association between NT1 and human leukocyte antigen (*HLA*)-DQB1^*^06:02

A strong association between NT1 and a specific HLA is well known (Table 1). In 1984, Honda and Juji found that all narcoleptic patients with cataplexy carried HLA-DR2 at the serological level^17^. This finding was extended to the *HLA* genotype level and showed that >90% of East Asians, such as Japanese, Koreans, and Chinese, as well as Europeans with NT1 carried the HLA-DRB1^*^15:01-DQB1^*^06:02 haplotype^18–21^. Conversely, only 12–25% of individuals in these general populations had this haplotype. Because the DRB1^*^15:01 and DQB1^*^06:02 alleles are in almost complete linkage disequilibrium in these populations, which *HLA* allele has the primary effect cannot be determined. In Africans, NT1 is associated with other *DRB1-DQB1* haplotypes, such as DRB1^*^11:01-DQB1^*^06:02 and DRB1^*^15:03-DQB1^*^06:02^18^. A minor proportion of East Asian and European patients also carry other DRB1-DQB1^*^06:02 haplotypes^18^. The concordance of DQB1^*^06:02 in these patients indicates that HLA-DQB1 rather than HLA-DRB1 affects development of the disease. NT2 is also associated with DQB1^*^06:02. Thirty to 50% of patients with NT2 are DQB1^*^06:02-positive, and the frequency is significantly higher than that in the general population but is lower than that in NT1 (Table 1)^20,22–24^.

**Table 1:** Significant associations between *HLA* and narcolepsy

Allele	Disease	Minimum *P-*value	Odds ratio	Study population
HLA-DQB1^*^06:02	NT1	0	Almost all patients carry this allele	African, Chinese, European, Japanese, and Korean^18–21,25,26,28^
HLA-DQB1^*^03:01	NT1	2 × 10^−14^	1.3–2.9	African, Chinese, European, Japanese, and Korean^18–21,28^
HLA-DQB1^*^03:02	NT1	8 × 10^−9^	2.0	Japanese^25^
HLA-DQB1^*^05:01	NT1	8 × 10^−10^	0.26–0.56	European and Japanese^18,25,28,29^
HLA-DQB1^*^06:01	NT1	1 × 10^−6^	0.10–0.39	Japanese and Korean^18,20,25^
HLA-DQB1^*^06:03	NT1	5 × 10^−14^	0.02–0.27	Chinese and European^26,28,29^
HLA-DPB1^*^04:02	NT1	9 × 10^−17^	0.38–0.47	Chinese and European^28^
HLA-DPB1^*^05:01	NT1	7 × 10^−5^	1.1–1.4	Chinese, European, and Japanese^25,28^
HLA-DQB1^*^06:02	NT2	1 × 10^−7^	2.4–5.2	European, Japanese, and Korean^20,22–24^

DQB1^*^06:02 is essential in NT1 because almost all patients carry this allele. However, because DQB1^*^06:02 is also a relatively common allele in the general population, the presence of DQB1^*^06:02 is not sufficient for NT1 development. The λ value for *HLA* of narcoleptic patients was calculated to be 2–4^18^. This λ value is below the relative risk in first-degree family members. These data indicate that additional genetic factors are likely to be involved in genetic predisposition to NT1.

## Complex *HLA* associations with NT1

Most patients carry DQB1^*^06:02 on at least one chromosome. However, other *DQB1* alleles on the other chromosome also contribute to susceptibility or resistance to NT1^18^. When the odds ratios for NT1 were calculated for DQB1^*^06:02 heterozygotes and homozygotes, an increased risk of NT1 was observed in the DQB1^*^06:02 homozygotes across multiple populations^18,25^. The disease susceptibility differs among heterozygous DQB1^*^06:02/other *DQB1* allele combinations (Table 1)^18–21,25,26^. DQB1^*^03:01 and DQB1^*^03:02 confer susceptibility to NT1, whereas DQB1^*^06:01, DQB1^*^06:03, and DQB1^*^05:01 are protective against the disease. The *HLA* region includes genes coding HLA-A, -B, -C, and -DP molecules in addition to those coding HLA-DR and -DQ molecules. Recently, immune-related diseases have been reported to be associated with multiple *HLA* genes. For example, the strong association between several *HLA-DRB1* alleles and rheumatoid arthritis is well known. A logistic regression analysis performed to identify independent associations other than *HLA-DRB1* in the *HLA* region revealed that *HLA-B* and *-DPB1* alleles were significantly associated with rheumatoid arthritis^27^. Regarding NT1, logistic regression analyses controlling for the effect of the *HLA-DQB1* alleles were also conducted to identify other independent *HLA* associations. As a result, the susceptibility effects of DPB1^*^05:01 and protective effects of DPB1^*^04:02 were identified across multiple ethnic groups (Table 1)^25,28^. Subsequent analyses that considered alleles of *HLA* class II genes, such as *HLA-DQB1* and *-DPB1*, demonstrated an independent association for *HLA* class I gene alleles^28,29^. These results suggest that complex HLA interactions, including both HLA class I and II molecules, contribute to the development of NT1, although DQB1^*^06:02 is an essential condition. Indeed, a recent study using an animal model reported that cytotoxic CD8+ T cells but not Th1 CD4+ cells were able to induce a selective loss of orexin neurons^30^. Therefore, cytotoxic responses may also play a central role in NT1 pathogenesis because orexin neurons can express HLA class I molecules.

## The ASO3-adjuvanted pandemic A/H1N1 2009 (Pandemrix) vaccine and NT1

The strong association with *HLA* indicates immunological pathogenesis of NT1. The onset of NT1 symptoms occurs most commonly in the late spring, suggesting that NT1 may be triggered or accelerated by winter infections. An association between *Streptococcus pyogenes* and NT1 has been found^31,32^. Recently, a new environmental factor in the development of NT1 has been reported. In April 2009, the Centers for Disease Control and Prevention reported patients infected with the 2009 pandemic influenza A (H1N1) virus, and vaccines were developed to protect against this virus. An H1N1 ASO3-adjuvanted pandemic vaccine (Pandemrix) was used in Scandinavia and other European countries. In August 2010, a possible association between the onset of NT1 and exposure to Pandemrix in children and adolescents was observed in Finland and Sweden^33^. In other European countries, several epidemiological studies also supported an increased risk of NT1 following Pandemrix administration^34–38^. In addition, many patients exposed to Pandemrix carried DQB1^*^06:02. In China, an increase in NT1 onset in children (all DQB1^*^06:02-positive) was observed in the winter of 2010 following the H1N1 2009–2010 pandemic season^39^. These data suggest that an interaction between *HLA* and an immune trigger or accelerator strongly affects NT1 development.

## Autoantibodies in NT1

The strong association between NT1 and *HLA* suggests an autoimmune etiology. Several groups have attempted to identify specific autoantibodies against orexin and orexin receptors in NT1. However, the results were neither specific to the disease nor replicated in independent studies. Accordingly, definitive evidence was still lacking. However, in 2010, autoantibody titers against Tribbles homolog 2 (TRIB2), which is enriched in orexin neurons, were found to be higher in sera from patients with NT1 than in healthy individuals, patients with idiopathic hypersomnia, or patients with other neurological disorders^40^. The titers were significantly associated with a short disease duration and were highest early after disease onset^41^. Autoantibodies against TRIB2 were also assayed in Japanese subjects, and higher TRIB2 antibody titers were confirmed in patients with NT1^42^. Accordingly, immunization against TRIB2 was expected to trigger the loss of orexin neurons. However, the destruction of orexin neurons can conversely induce autoantibodies against TRIB2. Contrary to the expectation, higher TRIB2 antibody titers in patients appear to be a consequence rather than a trigger of destruction^43^.

## Genome-wide association studies (GWASs) of NT1

Over the past decade, sequencing of the human genome and the HapMap project have produced dramatic progress in our understanding of genetic contributions to common diseases. The HapMap project determined genotypes of single-nucleotide polymorphisms (SNPs) in the human genome and provided data to the public^44^. High-throughput SNP typing technologies have enabled genotyping of a million SNPs in the HapMap data and subsequent GWASs^45,46^. GWASs have successfully identified genetic variants associated with common diseases without any prior information about gene functions^47^. As of 2018, GWASs have revealed >60,000 unique SNP-trait associations according to the GWAS Catalog (https://www.ebi.ac.uk/gwas/home). Understanding the genetic basis of a disease provides insights into its pathogenesis and is expected to contribute to the development of personalized medicine and new treatments. Regarding narcolepsy, several groups have performed GWASs to identify novel genetic factors associated with NT1 (Table 2).

**Table 2:** GWASs of narcolepsy type 1 (NT1)

Chr	rs number	Gene	Effect allele	Minimum *P-*value	Odds ratio (95% CI)	Study population
1	rs7553711	*TNFSF4*	C	4 × 10^−8^	1.3 (1.2–1.5)	European^57^
3	rs3181077	*CCR1/CCR3*	T	6 × 10^−6^	1.6 (1.3–2.0)	Japanese^59^
7	rs2854536	*TCRB*	G	4 × 10^−8^	0.78 (0.71–0.85)^a^	Chinese and European^58^
10	rs10995245	*ZNF365*	A	1 × 10^−11^	1.2 (1.2–1.3)^a^	Chinese and European^58^
14	rs1154155	*TCRA*	C	3 × 10^−22^	1.5 (1.3–1.8) −1.9 (1.6–2.2)	Chinese, European, Japanese, and Korean^55^
15	rs34593439	*CTSH*	A	2 × 10^−8^	1.3 (1.2–1.5)	European^57^
19	rs2305795	*P2RY11*	A	6 × 10^−10^	1.3 (1.2–1.4)^a^	Chinese, European, Japanese, and Korean^56^
21	rs2834188	*IL10RB-IFNAR1*	A	2 × 10^−8^	0.77 (0.71–0.85)^a^	Chinese and European^58^
22	rs5770917	*CPT1B*	C	4 × 10^−7^	1.4 (1.0–2.0) −1.8 (1.4–2.3)	Japanese and Korean^48^

*Chr* chromosome, *CI* confidence interval

^a^Odds ratios were obtained from the Mantel–Haenszel test in each study

Our group performed a GWAS with replication for patients in a Japanese population (381 cases and 579 controls) and found that the SNP rs5770917 adjacent to *carnitine palmitoyltransferase 1B* (*CPT1B*) was associated with NT1 (*P* = 4 × 10^−7^)^48^. This association was confirmed in Korean patients (*P* = 0.03) (115 cases and 309 controls). An expression quantitative trait loci (eQTL) analysis showed that the risk allele of rs5770917 was significantly associated with a low *CPT1B* mRNA expression level. CPT1B is the rate-controlling enzyme of long-chain fatty acid β-oxidation. Long-chain acyl-coenzyme As (CoAs) are converted to long-chain acylcarnitines by CPT1B, which allows their transport into the mitochondria, where they are metabolized through β-oxidation. The serum carnitine fractions were measured in patients with NT1 and control subjects. The acylcarnitine levels were abnormally low in approximately 20% of patients but were within the normal range in all of the controls^49^. These data suggest that fatty acid β-oxidation may be altered in NT1. Mouse studies support this possibility. Juvenile visceral steatosis (JVS) mice exhibit primary systemic carnitine deficiency that is caused by a defect in renal carnitine reabsorption^50^. The symptoms of JVS mice are aggravated during fasting. When locomotor activity and sleep in JVS mice are examined during fasting, reduced locomotor activity and a higher frequency of fragmented wakefulness are observed^51^. Interestingly, the reduced locomotor activity in fasted JVS mice is recovered by administration of modafinil, which is a wakefulness-promoting agent that is used for narcolepsy treatment. A significant reduction in c-Fos-positive orexin neurons was also observed in the fasted JVS mice^51^. Another study showed that a l-carnitine-rich diet eliminated suppression of the *orexin* mRNA expression levels in JVS mice^52^. Taken together, the data suggest that l-carnitine supplementation may improve narcolepsy. Therefore, our group conducted a clinical trial in which l-carnitine was administered to patients with NT1 to assess the efficacy of oral l-carnitine as a treatment^53^. Although the sample size was small (30 subjects), the total time of dozing off during the daytime was decreased by l-carnitine administration compared with that of placebo treatment. In addition, a case report described a patient with NT2 who was successfully treated with oral l-carnitine^54^. Further studies with larger numbers of patients and long-term observations are required to confirm its efficacy. The data regarding *CPT1B* and the subsequent effects of l-carnitine illustrate the importance of GWASs, which may ultimately lead to novel treatments.

A GWAS of European samples showed that SNP rs1154155 (*P* = 3 × 10^−22^), which is located in the *T-cell receptor* (*TCR*) alpha locus, and SNP rs9648789 (*P* = 4 × 10^−8^), which is located in the *TCR* beta locus, are associated with NT1^55^. The associations were also replicated in East Asian samples. HLA class I and II molecules interact with the TCR to present peptides to T cells. When the strong association between HLA-DQB1^*^06:02 and NT1 is taken into consideration, a logical assumption is that DQ0602 molecules interact with a specific TCR associated with the risk genotypes of rs1154155 and rs9648789. However, the exact role of these SNPs remains unclear. No reports have described variants in the *TCR* alpha and beta loci that are significantly associated with other autoimmune and inflammatory diseases in a genome-wide manner except for pancreatitis. The reason that no other diseases are associated with this locus may be because of the strength of the HLA associations. Indeed, the effect size of HLA-DQB1^*^06:02 in NT1 is much higher than those of *HLA* alleles associated with other autoimmune and inflammatory diseases. Another possibility is that a unique immune-related pathway may be responsible for the pathological mechanism of NT1. Further studies are needed to elucidate the disease mechanisms underlying the *HLA* and *TCR* associations in NT1.

Other susceptibility genes have been found by GWASs, most of which are immune-related genes. SNP rs2305795 in *purinergic receptor P2Y11* (*P2RY11*) is associated with NT1 (*P* = 6 × 10^−10^) and is correlated with the *P2RY11* expression levels in CD8+ T cells and natural killer cells^56^. The disease susceptible allele of rs2305795 has a protective effect against ATP-induced cell death in CD8+ and CD4+ T cells and natural killer cells. Other GWASs identified *cathepsin H* (*CTSH*), *tumor necrosis factor superfamily member 4* (*TNFSF4*), *zinc-finger protein 365* (*ZNF365*), *interleukin 10 receptor, beta* (*IL10RB*), *interferon alpha and beta receptor subunit 1* (*IFNAR1*), and *C-C chemokine receptor 1/3* (*CCR1*/*CCR3*)^57–59^. These reports suggest that NT1 results from inflammation or autoimmune processes in the central nervous system. Our group evaluated *Ccr3* knockout mice to elucidate the role of CCR3 in development of the disease. Fragmented sleep patterns were observed in the *Ccr3* knockout mice during the light phase. Interestingly, the number of orexin neurons in the lateral hypothalamus was slightly but significantly decreased in the *Ccr3* knockout mice compared with those of the wild-type mice. Therefore, CCR3 may be involved in sleep/wake regulation through the orexin system.

## Rare NT1 variants

When orexin deficiency was first shown to result in NT1, variant screening of the prepro-orexin and orexin receptor genes was performed, as mentioned above. However, common variants of these genes were not significantly associated with the disease in most patients. Only a single severe and early-onset case with a predicted dominant pathogenic variant in the orexin gene was identified (p.Leu16Arg)^60^. This variant was not present in the general population. The amino-acid encoded by the variant is located in the poly-leucine hydrophobic core of the signal peptide. This patient exhibited cataplexy at 6 months of age and was HLA-DQB1^*^06:02-negative, although NT1 typically begins during adolescence and usually the patients are HLA-DQB1^*^06:02-positive. Orexin A was undetectable in the patient’s CSF. Functional analyses revealed impaired trafficking and processing of the orexin mutant peptide^60^.

Recently, advances in next-generation sequencing technologies have had a strong impact on human genetics. Sequencing of all protein coding regions of the human genome (exome sequencing) has been utilized to identify novel pathogenic variants causing rare Mendelian diseases. Exome sequencing of familial narcolepsy individuals identified a missense deleterious variant (p.Ser133Cys) in the *myelin oligodendrocyte glycoprotein* (*MOG*)^61^ (Table 3), whereas no control carried the variant. MOG is a key autoantigen for primary demyelination in multiple sclerosis^62^. A transfection experiment was performed using mutant MOG-green fluorescent protein (GFP) and wild-type MOG-GFP. Ectopic clustering of the mutated protein was observed in the cytoplasm, whereas the wild-type protein mainly showed perinuclear and membrane localization. These results suggest that abnormalities in oligodendrocytes and myelin may play an important role in development of narcolepsy. Another study used exome sequencing and reported rare missense variants in *P2RY11* in two narcolepsy families. *P2RY11* re-sequencing in sporadic narcoleptic patients revealed six additional missense variants^63^. Six of the eight missense variants significantly decreased P2Y11 signaling (Table 3). The frequencies of the six deleterious variants were <0.3% in the general population. A GWAS found a significant association between SNP rs2305795 in the *P2RY11* region and NT1 (*P* = 6 × 10^−10^)^56^. Therefore, common as well as rare variants in *P2RY11* affect NT1 pathogenesis.

**Table 3:** Rare missense variants associated with narcolepsy type 1 (NT1) and autosomal dominant cerebellar ataxia, deafness, and narcolepsy (ADCA-DN)

Chr	rs number	Amino-acid change	Gene	Conditions
6	rs387906655	p.Ser133Cys	*MOG*^61^	NT1 (familial)
17	rs104894574	p.Leu16Arg	*HCRT*^60^	NT1 (sporadic)
19	rs758982075	p.Arg96Cys	*P2RY11*^63^	NT1 (sporadic)
19	rs145284058	p.Glu186Lys	*P2RY11*	NT1 (familial)
19	rs75945356	p.Tyr261Cys	*P2RY11*	NT1 (sporadic)
19	rs147142449	p.Val272Met	*P2RY11*	NT1 (sporadic)
19	rs146829743	p.Arg307Trp	*P2RY11*	NT1 (sporadic)
19	N/A	p.Tyr321His	*P2RY11*	NT1 (familial)
19	rs397509391	p.Val606Phe	*DNMT1*^65^	ADCA-DN (familial)
19	rs397509393	p.Gly605Ala	*DNMT1*	ADCA-DN (familial)
19	rs397509392	p.Ala570Val	*DNMT1*	ADCA-DN (familial)

*Chr* chromosome

Autosomal dominant cerebellar ataxia, deafness, and narcolepsy (ADCA-DN) is a neurologic disorder characterized by adult onset of progressive cerebellar ataxia, sensory neuronal deafness, narcolepsy with cataplexy, and dementia^64^. Patients with ADCA-DN show low orexin A levels in the CSF but are HLA-DQB1^*^06:02-negative. Exome sequencing in four families with ADCA-DN revealed that three pathogenic variants in exon 21 of *DNA methyltransferase 1* (*DNMT1*) were present in all affected family members (p.Ala570Val, p.Gly605Ala, and p.Val606Phe)^65^ (Table 3) but were not found in the general population. DNMT1 is widely expressed and is responsible for maintaining methylation patterns after DNA replication. A genome-wide assessment of DNA methylation showed hypomethylation in CpGs, with 0–10% and 80–95% methylation in ADCA-DN patients^66^. This observation suggests that changes in DNA methylation patterns may play an important role in ADCA-DN development. Recently, our group carried out an epigenome-wide association study of narcolepsy and found that the top-ranked differentially methylated positions associated with narcolepsy were significantly more abundant in non-CpG island regions and that >95% of the sites were hypomethylated^67^. Changes in global DNA methylation patterns are associated not only with ADCA-DN but also with narcolepsy. Furthermore, low orexin A levels in the CSF are commonly seen in both ADCA-DN and narcolepsy patients. Therefore, these two diseases may have a shared pathogenesis. The DNA methylation changes may contribute to decreased orexin A levels in the CSF or destruction of orexin neurons.

## Essential hypersomnia (EHS)

In contrast to NT1, the genetic backgrounds of other central disorders of hypersomnolence have not been well elucidated, most likely due to difficulty with their diagnoses, their heterogeneity, and their low prevalence compared with those of NT1. The International Classification of Sleep Disorders, 3rd edition, describes NT2 and idiopathic hypersomnia as central disorders of hypersomnolence other than NT1, but diagnosis is often a challenge^68^. The number of sleep onset REM periods (SOREMPs) during a multiple sleep latency test (MSLT) is used to distinguish whether a patient has NT2 or idiopathic hypersomnia. A diagnosis of NT2 requires two or more SOREMPs during the MSLT, whereas idiopathic hypersomnia can be diagnosed if the patient has less than two SOREMPs. However, the number of SOREMPs during a MSLT can produce a false positive; indeed, a sleep cohort study in the general population observed multiple SOREMPs in 13.1% of males and 5.6% of females^69^.

In 1986, Honda et al. designated a group of patients exhibiting narcolepsy-like conditions that lacked cataplexy as EHS^70^. The diagnostic criteria for EHS are based on the following three clinical items: (1) recurrent daytime sleep episodes that occur basically every day over a period of at least 6 months; (2) an absence of cataplexy; and (3) the hypersomnia cannot be explained by another sleep disorder, medical or neurological disorder, mental disorder, medication use, or substance use disorder. These criteria do not require determination of the number of SOREMPs. Thus, we focused on patients with EHS. Based on the International Classification of Sleep Disorders, 3rd edition, EHS consists of NT2 and a portion of patients with idiopathic hypersomnia (i.e., without a long sleep time), suggesting that EHS is still a heterogeneous disease. Reduction in the heterogeneity of EHS is required before conducting a genetic study. EHS is associated with HLA-DQB1^*^06:02^71^. In addition, a polygenic analysis using GWAS data and an *HLA* analysis revealed that HLA-DQB1^*^06:02-positive EHS patients had a greater shared genetic background with NT1 patients than with HLA-DQB1^*^06:02-negative EHS patients^72^. Furthermore, the two EHS groups based on the presence or absence of HLA-DQB1^*^06:02 have different etiologies. HLA-DQB1^*^06:02-positive EHS and NT1 are associated with similar susceptibility genes. Therefore, we performed a GWAS to identify susceptibility genes for HLA-DQB1^*^06:02-negative EHS^73,74^, for which fewer genetic studies have been performed. After a replication study, one SNP (rs10988217, located in the *carnitine O-acetyltransferase* (*CRAT*) region) reached the genome-wide significance level. The risk allele of rs10988217 was significantly associated with an increased *CRAT* expression level in an eQTL analysis. CRAT is a key enzyme that plays an important role in metabolic pathways involved in control of the short-chain acyl-CoA/CoA ratio. This enzyme catalyzes the reversible conversion of short-chain acyl-CoA and carnitine to acylcarnitine and free CoA^75,76^. As mentioned above, *CPT1B* encodes a rate-limiting enzyme that is involved in β-oxidation of long-chain acyl-CoAs and is a susceptibility gene for NT1. The observation that genes related to energy metabolism are associated with both HLA-DQB1^*^06:02-negative EHS and NT1 is interesting, although a difference exists between short-chain and long-chain compounds. In future analyses, we need to examine whether patients with HLA-DQB1^*^06:02-negative EHS have abnormalities in metabolic pathways related to short-chain acyl-CoAs.

## References

[1] Aserinsky E, Kleitman N (1953). Regularly occurring periods of eye motility, and concomitant phenomena, during sleep. Science.

[2] Vogel G (1960). Studies in psychophysiology of dreams. III. The dream of narcolepsy. Arch. Gen. Psychiatry.

[3] American Academy of Sleep Medicine. *International Classification of Sleep Disorders* 3rd edn. (American Academy of Sleep Medicine, Darien, IL, 2014).

[4] Honda Y (1979). Census of narcolepsy, cataplexy and sleep life among teenagers in Fujisawa city. Sleep. Res..

[5] Tashiro T, Kanbayashi T, Iijima S, Hishikawa Y (1992). An epidemiological study on prevalence of narcolepsy in Japanese. J. Sleep. Res..

[6] Mignot E (1998). Genetic and familial aspects of narcolepsy. Neurology.

[7] Lavie P, Peled R (1987). Narcolepsy is a rare disease in Israel. Sleep.

[8] Khatami R (2004). Monozygotic twins concordant for narcolepsy-cataplexy without any detectable abnormality in the hypocretin (orexin) pathway. Lancet.

[9] Vyse TJ, Todd JA (1996). Genetic analysis of autoimmune disease. Cell.

[10] Silber MH, Krahn LE, Olson EJ, Pankratz VS (2002). The epidemiology of narcolepsy in Olmsted County, Minnesota: a population-based study. Sleep.

[11] Lin L (1999). The sleep disorder canine narcolepsy is caused by a mutation in the hypocretin (orexin) receptor 2 gene. Cell.

[12] Chemelli RM (1999). Narcolepsy in orexin knockout mice: molecular genetics of sleep regulation. Cell.

[13] Sakurai T (1998). Orexins and orexin receptors: a family of hypothalamic neuropeptides and G protein-coupled receptors that regulate feeding behavior. Cell.

[14] Sakurai T (2007). The neural circuit of orexin (hypocretin): maintaining sleep and wakefulness. Nat. Rev. Neurosci..

[15] Nishino S, Ripley B, Overeem S, Lammers GJ, Mignot E (2000). Hypocretin (orexin) deficiency in human narcolepsy. Lancet.

[16] Mignot E (2002). The role of cerebrospinal fluid hypocretin measurement in the diagnosis of narcolepsy and other hypersomnias. Arch. Neurol..

[17] Juji T, Satake M, Honda Y, Doi Y (1984). HLA antigens in Japanese patients with narcolepsy. All the patients were DR2 positive. Tissue Antigens.

[18] Mignot E (2001). Complex HLA-DR and -DQ interactions confer risk of narcolepsy-cataplexy in three ethnic groups. Am. J. Hum. Genet..

[19] Han F (2012). HLA-DQ association and allele competition in Chinese narcolepsy. Tissue Antigens.

[20] Hong SC (2007). DQB1*0301 and DQB1*0601 modulate narcolepsy susceptibility in Koreans. Hum. Immunol..

[21] Roh EY (2006). Association of HLA-DR and -DQ genes with narcolepsy in Koreans: comparison with two control groups, randomly selected subjects and DRB1*1501-DQB1*0602--positive subjects. Hum. Immunol..

[22] Andlauer O (2012). Predictors of hypocretin (orexin) deficiency in narcolepsy without cataplexy. Sleep.

[23] Mignot E, Hayduk R, Black J, Grumet FC, Guilleminault C (1997). HLA DQB1*0602 is associated with cataplexy in 509 narcoleptic patients. Sleep.

[24] Miyagawa T (2015). An association analysis of HLA-DQB1 with narcolepsy without cataplexy and idiopathic hypersomnia with/without long sleep time in a Japanese population. Hum. Genome Var..

[25] Miyagawa T (2015). New susceptibility variants to narcolepsy identified in HLA class II region. Hum. Mol. Genet..

[26] Hor H (2010). Genome-wide association study identifies new HLA class II haplotypes strongly protective against narcolepsy. Nat. Genet..

[27] Raychaudhuri S (2012). Five amino acids in three HLA proteins explain most of the association between MHC and seropositive rheumatoid arthritis. Nat. Genet..

[28] Ollila HM (2015). HLA-DPB1 and HLA class I confer risk of and protection from narcolepsy. Am. J. Hum. Genet..

[29] Tafti M (2014). DQB1 locus alone explains most of the risk and protection in narcolepsy with cataplexy in Europe. Sleep.

[30] Bernard-Valnet R (2016). CD8 T cell-mediated killing of orexinergic neurons induces a narcolepsy-like phenotype in mice. Proc. Natl. Acad. Sci. USA.

[31] Longstreth WT, Ton TG, Koepsell TD (2009). Narcolepsy and streptococcal infections. Sleep.

[32] Aran A (2009). Elevated anti-streptococcal antibodies in patients with recent narcolepsy onset. Sleep.

[33] Nohynek H (2012). AS03 adjuvanted AH1N1 vaccine associated with an abrupt increase in the incidence of childhood narcolepsy in Finland. PLoS ONE.

[34] Dauvilliers Y (2013). Increased risk of narcolepsy in children and adults after pandemic H1N1 vaccination in France. Brain.

[35] Heier MS (2013). Incidence of narcolepsy in Norwegian children and adolescents after vaccination against H1N1 influenza A. Sleep. Med..

[36] Miller E (2013). Risk of narcolepsy in children and young people receiving AS03 adjuvanted pandemic A/H1N1 2009 influenza vaccine: retrospective analysis. BMJ.

[37] O’Flanagan D (2014). Investigation of an association between onset of narcolepsy and vaccination with pandemic influenza vaccine, Ireland April 2009-December 2010. Eur. Surveill..

[38] Stowe J (2016). Risk of narcolepsy after AS03 adjuvanted pandemic A/H1N1 2009 influenza vaccine in adults: a Case-Coverage Study in England. Sleep.

[39] Han F (2011). Narcolepsy onset is seasonal and increased following the 2009 H1N1 pandemic in China. Ann. Neurol..

[40] Cvetkovic-Lopes V (2010). Elevated Tribbles homolog 2-specific antibody levels in narcolepsy patients. J. Clin. Invest..

[41] Kawashima M (2010). Anti-Tribbles homolog 2 (TRIB2) autoantibodies in narcolepsy are associated with recent onset of cataplexy. Sleep.

[42] Toyoda H (2010). Anti-Tribbles homolog 2 autoantibodies in Japanese patients with narcolepsy. Sleep.

[43] Tanaka, S. et al. Anti-Tribbles Pseudokinase 2 (TRIB2)-immunization modulates hypocretin/orexin neuronal functions. *Sleep* **40**, (2017). 10.1093/sleep/zsw036 28364459

[44] The International HapMap Consortium. (2005). A haplotype map of the human genome. Nature.

[45] Matsuzaki H (2004). Parallel genotyping of over 10,000 SNPs using a one-primer assay on a high-density oligonucleotide array. Genome Res..

[46] Oliphant A, Barker DL, Stuelpnagel JR, Chee MS (2002). BeadArray technology: enabling an accurate, cost-effective approach to high-throughput genotyping. Biotechniques.

[47] The Wellcome Trust Case Control Consortium. (2007). Genome-wide association study of 14,000 cases of seven common diseases and 3,000 shared controls. Nature.

[48] Miyagawa T (2008). Variant between CPT1B and CHKB associated with susceptibility to narcolepsy. Nat. Genet..

[49] Miyagawa T (2011). Abnormally low serum acylcarnitine levels in narcolepsy patients. Sleep.

[50] Kuwajima M (1991). Animal model of systemic carnitine deficiency: analysis in C3H-H-2 degrees strain of mouse associated with juvenile visceral steatosis. Biochem. Biophys. Res. Commun..

[51] Yoshida G (2006). Fasting-induced reduction in locomotor activity and reduced response of orexin neurons in carnitine-deficient mice. Neurosci. Res..

[52] Kuwajima M (2007). Reduced carnitine level causes death from hypoglycemia: possible involvement of suppression of hypothalamic orexin expression during weaning period. Endocr. J..

[53] Miyagawa T (2013). Effects of oral L-carnitine administration in narcolepsy patients: a randomized, double-blind, cross-over and placebo-controlled trial. PLoS ONE.

[54] Romigi A (2015). Oral L-carnitine as treatment for narcolepsy without cataplexy during pregnancy: a case report. J. Neurol. Sci..

[55] Hallmayer J (2009). Narcolepsy is strongly associated with the T-cell receptor alpha locus. Nat. Genet..

[56] Kornum BR (2011). Common variants in P2RY11 are associated with narcolepsy. Nat. Genet..

[57] Faraco J (2013). ImmunoChip study implicates antigen presentation to T cells in narcolepsy. PLoS. Genet..

[58] Han F (2013). Genome wide analysis of narcolepsy in China implicates novel immune loci and reveals changes in association prior to versus after the 2009 H1N1 influenza pandemic. PLoS. Genet..

[59] Toyoda, H. et al. A polymorphism in CCR1/CCR3 is associated with narcolepsy. *Brain Behav. Immun*. **49**, 148–155 (2015). 10.1016/j.bbi.2015.05.003 25986216

[60] Peyron C (2000). A mutation in a case of early onset narcolepsy and a generalized absence of hypocretin peptides in human narcoleptic brains. Nat. Med..

[61] Hor H (2011). A missense mutation in myelin oligodendrocyte glycoprotein as a cause of familial narcolepsy with cataplexy. Am. J. Hum. Genet..

[62] Reindl M (1999). Antibodies against the myelin oligodendrocyte glycoprotein and the myelin basic protein in multiple sclerosis and other neurological diseases: a comparative study. Brain.

[63] Degn M (2017). Rare missense mutations in P2RY11 in narcolepsy with cataplexy. Brain.

[64] Melberg A (1995). Autosomal dominant cerebellar ataxia deafness and narcolepsy. J. Neurol. Sci..

[65] Winkelmann J (2012). Mutations in DNMT1 cause autosomal dominant cerebellar ataxia, deafness and narcolepsy. Hum. Mol. Genet..

[66] Kernohan KD (2016). Identification of a methylation profile for DNMT1-associated autosomal dominant cerebellar ataxia, deafness, and narcolepsy. Clin. Epigenetics.

[67] Shimada, M., Miyagawa, T., Toyoda, H., Tokunaga, K. & Honda, M. Epigenome-wide association study of DNA methylation in narcolepsy: an integrated genetic and epigenetic approach. *Sleep* **41**, (2018). 10.1093/sleep/zsy019 29425374

[68] Baumann CR (2014). Challenges in diagnosing narcolepsy without cataplexy: a consensus statement. Sleep.

[69] Mignot E (2006). Correlates of sleep-onset REM periods during the Multiple Sleep Latency Test in community adults. Brain.

[70] Honda Y (1986). HLA-DR2 and Dw2 in narcolepsy and in other disorders of excessive somnolence without cataplexy. Sleep.

[71] Miyagawa T (2009). Polymorphism located between CPT1B and CHKB, and HLA-DRB1*1501-DQB1*0602 haplotype confer susceptibility to CNS hypersomnias (essential hypersomnia). PLoS ONE.

[72] Yamasaki Maria, Miyagawa Taku, Toyoda Hiromi, Khor Seik-Soon, Liu Xiaoxi, Kuwabara Hitoshi, Kano Yukiko, Shimada Takafumi, Sugiyama Toshiro, Nishida Hisami, Sugaya Nagisa, Tochigi Mamoru, Otowa Takeshi, Okazaki Yuji, Kaiya Hisanobu, Kawamura Yoshiya, Miyashita Akinori, Kuwano Ryozo, Kasai Kiyoto, Tanii Hisashi, Sasaki Tsukasa, Honda Yutaka, Honda Makoto, Tokunaga Katsushi (2016). Evaluation of polygenic risks for narcolepsy and essential hypersomnia. Journal of Human Genetics.

[73] Khor SS (2013). Genome-wide association study of HLA-DQB1*06:02 negative essential hypersomnia. PeerJ.

[74] Miyagawa, T. et al. A variant at 9q34.11 is associated with HLA-DQB1*06:02 negative essential hypersomnia. *J. Hum. Genet*. **63**, 1259–1267 (2018). 10.1038/s10038-018-0518-8 30266950

[75] Jogl G, Tong L (2003). Crystal structure of carnitine acetyltransferase and implications for the catalytic mechanism and fatty acid transport. Cell.

[76] Miyata Y, Shimomura I (2013). Metabolic flexibility and carnitine flux: the role of carnitine acyltransferase in glucose homeostasis. J. Diabetes Investig..

